# Process Improvement by Eliminating Mixing of Whole Blood Units after an Overnight Hold Prior to Component Production Using the Buffy Coat Method

**DOI:** 10.1155/2013/154838

**Published:** 2013-06-05

**Authors:** Cherie Mastronardi, Peter Schubert, Elena Levin, Varsha Bhakta, Qi-Long Yi, Adele Hansen, Tamiko Stewart, Craig Jenkins, Wanda Lefresne, William Sheffield, Jason P. Acker

**Affiliations:** ^1^Canadian Blood Services, 1800 Alta Vista Drive, Ottawa, ON, K1G 4J5, Canada K1G 4J5; ^2^Canadian Blood Services, Centre for Blood Research, 2350 Health Sciences Mall, University of British Columbia, Vancouver, BC, Canada V6T 1Z3; ^3^Canadian Blood Services Research and Development, McMaster University, HSC 4N66, 1200 Main Street West, Hamilton, ON, Canada L8N 3Z5; ^4^Canadian Blood Services Research and Development, 8249 114th Street, Edmonton, AB, Canada T6G 2R8; ^5^Canadian Blood Services, Network Centre for Applied Development, Suite 207, 2150 Western Parkway, Vancouver, BC, Canada V6T 1V6

## Abstract

The elimination of a thorough manual mixing of whole blood (WB) which takes place following the overnight hold, but before the first centrifugation step, during buffy coat component production at Canadian Blood Services (CBS) was investigated. WB was pooled after donation and split. Pairs of platelet, red blood cell (RBC), and plasma components were produced, with half using the standard method and half using a method in which the mixing step was eliminated. Quality assessments included yield, pH, CD62P expression and morphology for platelets, hemoglobin, hematocrit, hemolysis, and supernatant K^+^ for RBCs, and volume and factor VIII activity levels for plasma. All components, produced using either method, met CBS quality control criteria. There were no significant differences in platelet yield between components produced with and without mixing. A significant difference was seen for RBC hemolysis at expiry (*P* = 0.03), but for both groups, levels met quality control requirements. Noninferiority of components produced without mixing was confirmed for all parameters. Manual mixing is laborious and has a risk of repetitive strain for production staff and its significance is unclear. Elimination of this step will improve process efficiencies without compromising quality.

## 1. Introduction

Whole blood (WB) collection and storage and processing conditions can have critical consequences not only for the immediate quality of the resulting components but also for their long-term quality during storage. At Canadian Blood Services (CBS), production of pooled platelet components (PCs) is a semiautomated process in which WB is held overnight and then separated by a hard centrifugation step into RBCs, plasma, and buffy coats (BCs), from which PCs are subsequently produced [1, 2]. CBS current practice includes a thorough, manual mixing of WB units following the overnight hold immediately prior to the first centrifugation step. In consultation with the bag vendors, this step was introduced during the implementation of semiautomated component production at CBS [1] and was thought to improve the separation of platelets from the other components in blood and hence results in a higher platelet yield. 

In contrast with other mixing steps, such as the proper mixing of WB with anticoagulant at donation [3, 4], there is no evidence regarding the benefits of mixing WB immediately prior to centrifugation. Mixing of WB units does not appear to have been investigated previously nor does it appear in most descriptions of component production methods found in the literature, yet desired platelet yields are still reported [5–8]. The aim of this study was to examine the impact of eliminating this nonevidence based step in our component production process, as it is labour intensive, time consuming, and has the additional disadvantage of creating a risk of repetitive strain injury for production staff.

## 2. Materials and Methods

### 2.1. Whole Blood Collection, Pooling, and Splitting

Ethical approval for this study was granted by CBS Research Ethics Board. The study was designed as a noninferiority study which used a pool and split design to generate homogenous starting samples. WB donations were collected from consenting healthy blood donors into 70 mL citrate-phosphate-dextrose anticoagulant. Following a minimum 3-hour rest period on cooling trays at room temperature (18–24°C), eight or nine ABO-matched units were docked together using sterile tubing with multiple connectors (MacoPharma) and an automated Compodock (Fresenius-Kabi, Bad Homburg, Germany) and drained into a 5 L pooling bag (waste bag from RBC Cell Wash Set, Haemonetics, Braintree, MA). Following gentle but thorough mixing, the pool was split back into the original blood bags in random order, with volumes based on the net weight of the WB pool (minus 3% for losses incurred during pooling and splitting) divided by the number of units in the pool. WB units were prelabeled either mixed (M) or nonmixed (NM) and were stored for approximately 8 h at room temperature prior to component production the following day.

### 2.2. Component Production

WB units from each pool were processed into leukoreduced pooled PCs in plasma, leukoreduced RBCs in saline-adenine-glucose-mannitol preservative, and frozen plasma following the standard semiautomated BC production method currently in place at CBS, as described previously [1, 2]. The 4 WB units labeled M were manually and thoroughly mixed prior to the first centrifugation step, while those labeled NM were not. A total of 12 WB pools were used for the study, each of which generated 2 pooled PCs (1 M, 1 NM), 8 RBC components (4 M, 4 NM), and 6 plasma components (3 M, 3 NM).

### 2.3. Sampling and Testing of PCs

PCs were sampled on Day 1 and Day 5 and were tested on Day 1 (all tests), Day 5 (for CD62P, extent of shape change (ESC) and morphology), and after expiry (Days 7-8 of storage for pH and platelet yield). Unit weight was used to calculate volume based on a specific gravity of 1.03 g/mL. Calculations for platelet yield on Day 1 and after expiry were made using this calculated volume. A total of 24 PCs was tested (12 M, 12 NM).

#### 2.3.1. CBS QC Requirements for PCs

Platelet yield (platelet count/unit) was calculated using the component volume and the platelet count, measured on an ADVIA 120 Hematology Analyzer (Siemens, Mississauga, ON, Canada), as described previously [1]. pH was measured using a GEM premier 3000 blood gas analyzer (Instrumentation Laboratory, Orangeburg, NY) and corrected to 22°C or a Thermo Orion 3-Star pH Meter (ThermoFisher Scientific, Nepean, Ontario, Canada). Residual leukocyte count was measured on the day of production using a flow cytometric assay as described previously [9], and sterility was tested on the day of production and at expiry using the BacT/ALERT system (automated BacT/ALERT 3D system, bioMérieux Canada Inc., St Laurent, Quebec, Canada) as described previously [10].

#### 2.3.2. Platelet Quality Assays

Assessment of platelet degranulation, a marker of platelet activation, was conducted using CD62P expression by flow cytometry on a FACS Canto II (BD, Mississauga, Ontario, Canada), as described previously [1, 11]. Platelet morphology was assessed by modified Kunicki scoring, as described previously [1, 11, 12]. ESC was measured using a SPA-2000 (Chronolog Corp., Havertown, PA) according to Holme et al. [13] and described in detail previously [1]. 

### 2.4. Sampling and Testing of RBC Components

RBCs were tested at Day 3 and Day 43 after collection. Unit volume was determined based on the net RBC unit weight and a specific gravity of 1.06 g/mL. Components were sampled and prepared for residual leukocyte count within 24 hours of production. In total, forty (20 M/20NM) RBCs were tested.

#### 2.4.1. CBS QC Requirements for RBCs

Total hemoglobin (g/L) was measured on an ADVIA 120 automated hematology analyzer and g/unit determined using the calculated volume of the RBC unit. RBCs were tested for rWBC levels using a flow cytometric approach as described previously [9] and were tested after expiry for sterility using the BacT/ALERT system. RBCs were tested for hemolysis levels using two methods. For the manual Drabkin's method, hematocrit (Hct) was determined visually following centrifugation in a Hettich 2010 microhematocrit centrifuge (Hettich Lab Technology, Tuttlingen, Germany) at 12,800 RPM. Supernatant and total hemoglobin were measured using the Drabkin's method as described previously [14], with trilevel commercial hemoglobin controls (Stanbio Laboratories, Boerne, TX) used for quality control. For automated hemolysis determination, Hb_T_ and Hct were determined using the ADVIA 120 automated hematology analyzer. Hb_S_ levels were measured using a HemoCue Plasma/Low automated hemoglobin analyzer (HemoCue AB, Ängelholm, Sweden). For both methods, percentage hemolysis was calculated using the following equation:

(1) $$\begin{array}{c} \%\;\text{hemolysis}=\left[\left(100-\text{Hct}\right)\times {\text{Hb}}_{\text{S}}\right]/{\text{Hb}}_{\text{T}}. \end{array}$$

#### 2.4.2. Additional RBC Quality Testing

Extracellular K^+^ concentrations were determined by indirect potentiometry using ion selective electrodes on a Beckman Coulter DXC 800 (Beckman Coulter Inc., Fullerton, CA).

### 2.5. Sampling and Testing of Plasma Components

The volume of 36 (18 M/18 NM) plasma components was measured. Plasma was placed into storage at ≤−20°C and 14 randomly selected components (7 M/7 NM) were shipped frozen for FVIII activity testing within 8 weeks of production, as described previously [15, 16]. 

### 2.6. Data Analysis

Two approaches were applied. Firstly, it was determined whether all components met acceptable QC criteria based on the CBS standard panel of QC tests (Table 1). Some tests were conducted both early and late in storage to determine whether quality was affected immediately after processing. Secondly, all QC parameters were assessed to determine whether components produced without mixing were noninferior to those produced using the standard protocol, based on noninferiority/equivalence margins, determined by reviewing one year of CBS QC data and data from an on-going internal Quality Monitoring Program (QMP) [11, 15]. Additional quality parameters were also measured, and if there were sufficient prior QMP data (CD62P expression for platelets and supernatant K^+^ for RBCs), noninferiority margins were determined and applied (Table 1). Sample size calculations assumed a significance level of 5% and a power of 80% (Table 1). 

The data are clustered for each WB pool from which multiple components of the same type and group (i.e., M or NM) were produced. A paired *t*-test was used to confirm whether the difference between the two groups was statistically significant. To confirm noninferiority of the nonmixing process, the difference between the two groups and the 95% confidence interval was calculated. If the upper and/or lower bounds of the 95% confidence interval were within the specified noninferiority margin, then noninferiority was confirmed. A two-sided *P* value of <0.05 was considered significant. 

## 3. Results

### 3.1. PCs

#### 3.1.1. Quality Control of PCs

There was no significant difference in volume between pooled PCs produced with or without mixing (334 ± 10.6 mL versus 334 ± 10.1 mL, resp.), indicating that pooling and splitting produced uniform components. Both the M and NM components produced from one pool did not meet QC criteria for platelet yield after expiry, possibly due to low donor platelet counts in that specific WB pool (Table 2). Otherwise, all PCs produced from M or NM blood met current QC acceptance criteria, and no bacteria were found in any unit.

#### 3.1.2. Comparison of PC Quality

Significant differences between M and NM groups were not observed, and noninferiority of the NM samples was confirmed for rWBC count on Day 1 and platelet yield and pH both on Day 1 and after expiry (Table 2). Using previous QMP data [11], a noninferiority margin of 7% was determined for CD62P expression (Table 1). Both on Day 1 and at expiry, no significant difference in CD62P expression between M and NM platelets was observed (Table 2). Similar to CD62P expression, ESC and morphology are not part of the routine QC panel but are often used as a means to assess platelet quality [11, 13], although there was insufficient QMP data to determine noninferiority margins (Table 2). No significant differences in morphology between NM and M platelets at Day 1 or Day 6 were seen. A significant difference in ESC between the 2 arms was observed at Day 1 (*P* < 0.01) with NM pooled platelets displaying lower ESC compared to M pooled platelet products. No difference between M and NM groups was seen at expiry (Table 2).

### 3.2. RBCs

#### 3.2.1. Quality Control of RBC Components

For RBCs, there was also no significant difference in volume between components produced from whole blood units with or without mixing (277 ± 3.6 mL versus 277 ± 3.8 mL, resp.). Bacterial growth was not detected in any unit, and all QC criteria were met with 100% pass rates for all parameters for both M and NM samples (Table 3). 

#### 3.2.2. Comparison of RBC Component Quality

No significant difference was observed for rWBCs/unit between M and NM groups (Table 3). Total hemoglobin levels on Day 3 and Day 43 were similar between the two arms, and no significant differences in Hct were seen. Based on the predetermined margins for rWBCs/unit, hemoglobin, and Hct, noninferiority of RBCs produced from WB that was not mixed was confirmed (Table 3). Hemolysis was measured using the CBS standard QC HemoCue/ADVIA method, and no significant differences were observed between M and NM groups at Day 3. A significant difference was seen at expiry (*P* = 0.03), with the NM units showing greater hemolysis. Despite this, noninferiority was confirmed, as the upper bound of 95% confidence interval of 0.029% was less than the margin (0.06%).

Measurement of hemolysis using the Drabkin method showed no significant differences between the M and NM arms and noninferiority was again confirmed (Table 3). There was a significant difference in supernatant K^+^ between the 2 arms at Day 3 (*P* = 0.02) with higher supernatant K^+^ observed in the NM units, but no difference at Day 43. Early in storage, levels of supernatant K^+^ are low (~5 mM), and they rise steadily during hypothermic storage [17]. Therefore, two noninferiority margins were determined and applied, one based on levels early in storage and one based on levels after expiry. Noninferiority of the NM units was confirmed at both time points (Table 3).

### 3.3. Plasma Components

Volumes were not significantly different between plasma produced from M or NM WB (294 ±5.8 mL versus 295 ± 6.0 mL, resp., *P* = 0.08), and all units passed QC requirements for sterility. FVIII activity was examined as this is one of the most labile of the coagulation factors, is often used as an indicator of plasma quality [16], and is a regulated QC requirement in Canada (Table 1). FVIII activity levels were 0.79 ± 0.10 IU/mL and 0.78 ± 0.12 IU/mL for M and NM components, respectively, with no significant difference found (*P* = 0.41). Noninferiority of the NM units was confirmed.

## 4. Discussion

Many aspects of the processes used to produce components from WB have been intensively investigated, including collection and storage bags [18], the anticoagulant used for WB collection, the importance of adequate mixing of the WB and anticoagulant at donation [19, 20], and the optimal time and temperature of WB storage prior to component production [21, 22]. However, there are elements of the process that are nonevidence based, and this study assessed one such step to determine whether it is value added or has any effect on product quality. Elimination of the mixing step produced acceptable products as all QC criteria were met. For all parameters tested, noninferiority criteria were met, indicating that components produced without WB mixing prior to centrifugation are not inferior to those produced with mixing. For some *in vitro* quality tests, differences were found between the M and NM groups. Early in storage, there were differences between the two arms for platelet ESC, with lower ESC, indicating less responsive platelets [13, 23], seen in the NM platelets; however, these differences disappeared when platelets were tested at expiry. Although lower ESC was seen in the NM group, both M and NM groups displayed responsiveness of greater than 30%, indicating overall good quality. 

Similarly for RBCs, supernatant potassium levels were found to be significantly different between the M and NM groups at Day 3 of storage, a difference also not seen at expiry. The levels seen between the groups early in storage differ by just 0.1 mmol/L, and although statistically significant, this difference should not affect product quality. A significant difference between M and NM RBCs was also observed for hemolysis measured using the standard HemoCue/ADVIA method used for QC at CBS but not using the Drabkin's method. Levels of hemolysis measured early in storage and at expiry using the Drabkin's method were higher than those measured using the HemoCue/ADVIA method. This underestimation of hemolysis by the HemoCue/ADVIA method has been observed before [24], and the differences in measurement of hemolysis using different methods are well documented [14, 25].

Communication with a number of blood centres in Europe and Australia which use a similar component production method to CBS reveals that some mix and some do not, but all achieve their desired platelet yields (personal communication with Dr. Denese Marks, Australian Red Cross, Australia; Gordon Nicholson, National Health Service Blood and Transplant England; Dr. Pieter van der Meer, Sanquin, The Netherlands and Dr. Alex Morrison, Scottish National Blood Transfusion Service, Scotland). Among the centres we contacted, none have transitioned from a process that mixed WB prior to centrifugation to one that did not, and so we are not aware of any validation or investigation of the effect of this process change. The results of the current study demonstrate that elimination of the mixing step is not detrimental to product quality, and components produced without mixing of the WB will meet all necessary QC criteria. In terms of process efficiency, and also for the health and safety of the production staff processing the blood, removal of this step may indeed be beneficial, and at CBS we are currently planning to implement this change.

## Figures and Tables

**Table 1:** Quality control acceptance criteria, noninferiority margins, and testing time points for platelet, RBC, and plasma components.

Parameter (unit)	Testing point	QC acceptance criteria	Noninferiority margin*	Sample size (pairs)^†^	Number of pairs tested^‡^
Platelets					

Platelet yield/unit (×10^9^/unit)	Day 1 and at expiry	>240 in 75% of units at expiry	50	12	12
pH	Day 1 and at expiry	6.4–7.8 in 100% of units at expiry	0.2	4	
rWBC (×10^6^/unit )	Day 1	<5 in 100% of units	0.05	4	
Sterility (growth/no growth)	Day 1 and at expiry	No growth in 100% of units	N/A	N/A	
Platelet CD62P (%)	Day 1 and Day 5	N/A	7	10	

RBC					

Hematocrit (L/L)	Day 3 and Day 43	0.5–0.7 in 90% of units	0.03	11	20
Hemoglobin (g/unit)	Day 3 and Day 43	≥35 g/unit (in 100%) & ≥40 g/unit (in 90% of units)	5	11	
rWBC (×10^6^/unit)	Day 1	<5 in 100% of units	1	20	
Hemolysis %	Day 3 and Day 43	<0.8 in 100% of units at expiry	0.06	11	
Sterility (growth/no growth)	At expiry	No growth in 100% of units	N/A	N/A	
Supernatant potassium (mmol/L)	Day 3 and Day 43	N/A	1.3/4^§^	9	

Plasma					

Factor VIII (IU/mL)	<8 weeks after production	>0.52 in 75% of units	0.3	7	7
Volume (mL)	<8 weeks after production	≥100	10	18	20
Sterility (growth/no growth)	<8 weeks after production	No growth in 100% of units	N/A	N/A	

*Noninferiority margins were determined using previous CBS quality control and quality monitoring program data.

^†^Sample size calculations assumed a significance level of 5% and a power of 80%.

^‡^The number of paired samples (mixed/not mixed) tested for each component type equals the largest sample size required for each component type, except for plasma, for which 7 pairs were tested for FVIII activity and 20 pairs were used to assess volume.

^§^As supernatant potassium levels increase during storage, noninferiority margins were determined based on levels early in storage (Day 3—1.3 mM) and after expiry (Day 43—4 mM).

N/A: not applicable and rWBC: residual white blood cells.

**Table 2:** Quality of pooled platelets produced with and without mixing. Sample size was 12 pairs (mixed/not mixed) for all parameters on Day 1 and after expiry. On Day 5, sample size was 10 pairs for CD62P and 11 pairs for morphology. In keeping with current QC processes at CBS, rWBC/unit was assessed at the beginning of storage, and platelet yield/unit and pH were tested after expiry. All QC criteria (parameters in bold) were met and noninferiority of platelets produced without being mixed was confirmed.

Testing time	Parameter	Study arm	Mean ± SD	Passed QC? (pass rate)	Difference (95% CI)
Day 1	**rWBC/unit**	NM	3.40 × 10^4^ ± 1.78 × 10^3^	Yes (100%)	−0.000 (−0.001, 0.001)
		M	3.37 × 10^4^ ± 1.32 × 10^3^	Yes (100%)	
	Platelet yield/unit (×10^9^/unit)	NM	256 ± 36.4	N/A	−0.08 (−8.87, 8.70)
		M	256 ± 28.3	N/A	
	pH (corrected to 22°C)	NM	6.93 ± 0.03	N/A	0.02 (−0.01, 0.06)
		M	6.95 ± 0.05	N/A	
	CD62P (% of population)	NM	15.71 ± 4.06	N/A	−0.19 (−2.28, 1.90)
		M	15.53 ± 3.78	N/A	
	Morphology	NM	358 ± 8.78	N/A	−0.29 (−7.89, 7.31)
		M	358 ± 12.39	N/A	
	Extent of shape change (%)	NM	37.7 ± 3.07	N/A	2.85 (1.22, 4.47)
		M	40.5 ± 3.78	N/A	

Day 5	CD62P (% of population)	NM	26.00 ± 3.53	N/A	1.169 (−0.021, 2.358)
		M	27.17 ± 4.28	N/A	
	Morphology	NM	327 ± 7.0	N/A	−4.864 (−11.150, 1.423)
		M	322 ± 8.9	N/A	
	Extent of shape change (%)	NM	36.2 ± 5.52	N/A	0.588 (−1.757, 2.932)
		M	36.8 ± 5.25	N/A	

After expiry	**Platelet yield/unit (×**10^9^ **/unit)**	NM	279 ± 29.3	Yes (91.6%)	−0.667 (−10.920, 9.587)
		M	278 ± 27.4	Yes (91.6%)	
	**pH (corrected to 22**°**C)**	NM	7.38 ± 0.058	Yes (100%)	0.033 (−0.006, 0.073)
		M	7.42 ± 0.058	Yes (100%)	

SD: standard deviation, QC: quality control, CI: confidence interval, NI: noninferiority, rWBC: residual white blood cells, M: mixed, NM: not mixed, and N/A: not applicable.

**Table 3:** Quality of red cell concentrates produced with and without mixing. Sample size was 20 pairs (mixed/not mixed) for all parameters. All QC criteria (parameters in bold) were met and noninferiority of RBCs produced without mixing was confirmed for all parameters.

Testing time	Parameter	Study arm	Mean ± SD	Passed QC? (pass rate)	Difference (95% CI)
Day 3	**rWBC/unit**	NM	1.18 × 10^5^ ± 7.11 × 10^4^	Yes (100%)	0.89 (−3.62, 5.41) × 10^4^
		M	1.27 × 10^5^ ± 9.96 × 10^4^	Yes (100%)	
	**Hemoglobin ** **(g/unit)**	NM	52.5 ± 1.53	Yes (100%)	−0.061 (−0.499, 0.376)
		M	52.4 ± 1.40	Yes (100%)	
	**Hematocrit (L/L)**	NM	0.626 ± 0.009	Yes (100%)	−0.002 (−0.006, 0.002)
		M	0.624 ± 0.008	Yes (100%)	
	Hemolysis (%): hemoCue/ADVIA	NM	0.045 ± 0.042	N/A	−0.011 (−0.029, 0.006)
		M	0.033 ± 0.023	N/A	
	Hemolysis (%): Drabkin's	NM	0.084 ± 0.019	N/A	0.001 (−0.007, 0.008)
		M	0.084 ± 0.021	N/A	
	Supernatant potassium (mM)	NM	5.830 ± 0.207	N/A	−0.095 (−0.175, −0.015)
		M	5.735 ± 0.220	N/A	

Day 43	**Hemoglobin ** **(g/unit)**	NM	52.3 ± 1.47	Yes (100%)	−0.128 (−0.575, 0.318)
		M	52.2 ± 1.64	Yes (100%)	
	**Hematocrit (L/L)**	NM	0.649 ± 0.011	Yes (100%)	−0.004 (−0.006, 0.002)
		M	0.646 ± 0.009	Yes (100%)	
	**Hemolysis (%): hemoCue/ADVIA**	NM	0.162 ± 0.044	Yes (100%)	0.016 (0.002, 0.029)
		M	0.178 ± 0.039	Yes (100%)	
	Hemolysis (%): Drabkin's	NM	0.198 ± 0.034	N/A	0.012 (−0.008, 0.032)
		M	0.210 ± 0.036	N/A	
	Supernatant potassium (mM)	NM	40.460 ± 1.898	N/A	−0.615 (−1.259, 0.029)
		M	39.845 ± 1.484	N/A	

SD: standard deviation, QC: quality control, CI: confidence interval, NI: noninferiority, rWBC: residual white blood cells, M: mixed, NM: not mixed, and N/A: not applicable.

## Abbreviations

BC: Buffy coat
CBS: Canadian blood services
ESC: Extent of shape change
Hct: Hematocrit
Hb_S_: Supernatant haemoglobin
Hb_T_: Total haemoglobin
M: Mixed
NM: Not mixed
PC: Platelet component
QC: Quality control
QMP: Quality monitoring program
RBC: Red blood cell
WB: Whole blood.
